# Acute Exacerbation of Idiopathic Pulmonary Fibrosis

**DOI:** 10.3390/medicina55030070

**Published:** 2019-03-16

**Authors:** Tomoo Kishaba

**Affiliations:** Department of Respiratory Medicine, Okinawa Chubu Hospital, 904-2293 Miyazato 281, Uruma City, Okinawa, Japan; kishabatomoo@gmail.com; Tel.: +81-98-973-4111

**Keywords:** acute exacerbation, consolidation, GGO, HRCT, idiopathic, IPF, LDH, nintedanib, pirfenidone, triggered

## Abstract

Idiopathic pulmonary fibrosis (IPF) is the most common form of idiopathic interstitial pneumonia. Idiopathic pulmonary fibrosis is often seen in elderly men who smoke. A diagnosis of IPF is based on a combination of a detailed clinical history, specific physical examination, laboratory findings, pulmonary function tests, high-resolution computed tomography (HRCT) of the chest, and histopathology. Idiopathic pulmonary fibrosis has a heterogeneous clinical course, from an asymptomatic stable state to progressive respiratory failure or acute exacerbation (AE). Acute exacerbation of IPF has several important differential diagnoses, such as heart failure and volume overload. The International Working Group project proposed new criteria for defining AE of IPF in 2016, which divides it into triggered and idiopathic AE. On the basis of these criteria, physicians can detect AE of IPF more easily. The recent international IPF guidelines emphasized the utility of chest HRCT. In addition, two antifibrotic agents have become available. We should focus on both the management and prevention of AE. The diagnostic process, laboratory findings, typical chest imaging, management, and prognosis of AE are comprehensively reviewed in this article.

## 1. Introduction

Idiopathic pulmonary fibrosis (IPF) is a chronic parenchymal lung disease with unknown etiology and is the most common fibrotic lung disease of all idiopathic interstitial pneumonias [1]. The majority of IPF patients are male, greater than 60 years old, and smokers [1,2]. Familial clustering is identified in approximately 3% of cases [3]. Genetic factors, such as *MUC5B*, are associated with the development of IPF [4,5]. The natural history of IPF is quite heterogeneous, from chronic stable symptoms to progressive respiratory failure or acute exacerbation (AE) [6]. The incidence of AE of IPF is 5–10% per year [7]. However, the incidence varies according to ethnicity. Japanese patients are more susceptible to AE of IPF [8]. Therefore, some genetic regulatory factors may be related to AE [9]. In addition, after the introduction of antifibrotic agents, the incidence of AE may be decreased. In this review, I will describe clinical pictures, laboratory findings, and chest imaging, especially high-resolution computed tomography (HRCT) findings, as well as the management and prognosis of AE of IPF.

## 2. Risk Factors

Studies have reported that reduced pulmonary function, especially forced vital capacity (FVC), never smoking status, and baseline serum Krebs von den Lungen-6 (KL-6) are crucial risk factors that predict an AE of IPF [10,11,12,13]. Reduced FVC patients often have decreased normal lung area due to extensive fibrosis. Patients with these characteristics are prone to developing severe lung injury that is consistent with gefitinib-associated interstitial lung disease (ILD) [14]. In the never smoking IPF patients, the baseline dyspnea grade and serial progression of dyspnea can predict the short-term development of AE [15]. Recently, Collard et al. [16] reported that baseline FVC, baseline supplemental oxygen, baseline antacid medication, and smoking are important risk factors for AE of IPF. Thus, once we diagnose IPF, serial physiological evaluations and reducing the impairment of daily activity due to AEs are the main tasks for physicians.

## 3. Diagnosis Process

The International Working Group Report proposed several revised criteria for an AE of IPF in 2016 [17]. The important background for a patient is a previous diagnosis of IPF and acute worsening or development of progressive dyspnea that has a duration of less than one month. Another important change is that bronchoalveolar lavage is no longer necessarily required for the diagnosis of AE compared with the 2007 criteria [18]. Therefore, AE of IPF can be diagnosed in a general hospital. These criteria provide rather broad coverage compared to the original 2007 criteria [7,17].

The new criteria divided AE of IPF into two groups, which are, namely, triggered and idiopathic. The triggered AE consists of episodes that are related to infection, drug toxicity, and aspiration, as well as those that occur after procedures and post-operatively. In terms of history, it is important that disease duration is less than 1 month. Furthermore, we should exclude pneumothorax and pleural effusion by chest radiography, as well as pulmonary embolism by a combination of clinical history and contrast enhanced chest CT. Furthermore, if we see bilateral infiltrates on plain chest radiographs, chest CTs will be required for the evaluation of bilateral ground-glass opacity (GGO), which will be consolidated with chronic fibrotic findings such as honeycombing, traction bronchiectasis/bronchiolectasis or reticular opacity. Before arriving at a diagnosis of AE of IPF, we should exclude heart failure or volume overload clinically.

In terms of physical findings, patients invariably exhibit acute respiratory distress, with prominent use of auxiliary muscles for respiration, especially the scalene muscles of the neck. Lung auscultation reveals bilateral diffuse fine crackles. Furthermore, IPF patients sometimes have clubbed fingernails.

## 4. Biomarkers

In laboratory findings, classic inflammatory markers such as white blood cell counts and C-reactive protein are usually elevated [1]. In the chemistry panel, lactate dehydrogenase (LDH) is a simple and sensitive marker that predicts the short-term prognosis of AE of IPF patients. Kishaba et al. [19] reported that the serial trend of serum LDH is associated with 90-day mortality of AE. In addition, Enomoto et al. [20] showed that a serum ferritin level above 500 ng/mL predicts poor prognosis in AE of IPF. Recently, serum periostin has received attention as a potentially attractive biomarker of IPF. Serum periostin is elevated both during the acute phase and the chronic stable phase in IPF patients [21,22]. Furthermore, serum decorin is a small, leucine-rich proteoglycan that has been introduced as a novel marker for AE of IPF. Nikaido et al. [23] reported that serum decorin had a significant association with oxygenation. Further multicenter studies will be required for further validation.

Both serum KL-6 and surfactant protein-D (SP-D) are useful markers of IPF [24,25]. However, KL-6 is a high-molecular weight protein. Therefore, the response of KL-6 is slower than that of LDH, and the elevation of serum KL-6 is commonly seen after the acute phase of AE. The elevation of serum SP-D reflects inflammatory processes, so this is often elevated in patients with severe pneumonia [26]. Therefore, distinguishing AE of IPF from severe pneumonia by serum SP-D alone is relatively difficult. 

## 5. Clinical Characteristics According to the New IPF Criteria

Based on the new 2016 proposed criteria, we reported that patients with triggered AE of IPF showed more extensive new shadow compared to those with idiopathic AE [19]. Multivariate analysis showed that the serum LDH and the serial trend of LDH predicted 90-day mortality [19]. The Kaplan–Meier survival curve of this study showed that a serum LDH level above 80 IU/L as measured within 2 weeks was associated with poor survival (*p* = 0.046) [19]. Yamazoe et al. [27] showed that patients with idiopathic AE were more likely to receive corticosteroids and more likely to develop AE during the winter months. On the contrary, the triggered group was more likely to have underlying lung cancer, compared to the idiopathic group (59.1% versus 7.1%, *p* < 0.001). In the idiopathic group, white blood cell and hemoglobin levels were independent predictors of in-hospital mortality [27]. Teramachi et al. [28] reported that AE of IPF accounted for approximately one-third of first hospitalizations for acute respiratory deterioration.

## 6. Imaging

In chest radiographs, a new bilateral diffuse shadow that is superimposed on the lower-lobe reticular shadow is the typical finding in AE of IPF patients. A comparison with previous films is a necessary first step in diagnosis.

The latest international IPF guidelines (2018) strongly insist upon the importance of chest HRCT for AE of IPF [1]. We should look for the coexistence of the usual interstitial pneumonia (UIP) pattern such as subpleural and basal predominant opacity, with peripheral dominant and heterogeneous distribution. Furthermore, there can be architectural distortion such as traction bronchiectasis and honeycombing [29]. Akira et al. [30] proposed that the CT findings of AEs of IPF should be divided into three patterns, consisting of peripheral, multifocal, and diffuse infiltrates. The prevalence of the new parenchymal shadow was significantly more frequent than the other two patterns. They also evaluated several follow up CT scans. In survivors with the peripheral pattern, the majority of GGO and consolidation regressed back to baseline levels of abnormality. In survivors with multifocal scan findings, GGO and consolidation disappeared with corticosteroid therapy. In contrast, survivors with the diffuse pattern demonstrated significant extension of GGO and consolidation. The Kaplan–Meier survival curve showed significant difference based on CT pattern [30]. In multivariate analysis, the diffuse CT pattern was the strongest predictor of mortality. In another study, Kishaba et al. [31] showed that the staging of AE of IPF is useful for the prediction of prognosis. Four important parameters were identified: serum LDH, KL-6, the ratio of partial pressure of oxygen to the fraction of inspiratory oxygen concentration, and the sum of the GGO and consolidation scores. They assigned points for each parameter and divided patients into two groups, with limited or extensive involvement. In addition, patients in the extensive group had poor prognoses compared to the limited group [31].

According to these studies, a detailed assessment of the chest HRCT findings in patients with AE of IPF can inform management and prognosis for physicians.

## 7. Management

The 2018 Japanese IPF treatment guidelines suggested that IPF patients with AE should be treated with corticosteroids, including pulse therapy [32]. Steroid pulse therapy is typically administered for three consecutive days. Weekly pulse therapy may sometimes be repeated once or twice. Prolonged pulse therapy may often be complicated by opportunistic infections such as pneumocystis pneumonia and viral infections. Therefore, the meticulous titration of prednisolone dosage is required during maintenance phase. Idiopathic pulmonary fibrosis itself is a fibrotic lung disease. However, there is a component of inflammation in AE of IPF [2]. Therefore, some patients respond to corticosteroids [33]. In addition, when we see a partial response with prednisolone, we commence treatment with chronic immunosuppressants, such as intravenous cyclophosphamide [34]. However, this treatment strategy is not supported by robust evidence. Recently, two novel therapies have been reported to have possible value in treating AEs of IPF. A recent report showed that direct hemoperfusion with a polymyxin B-immobilized fiber column (PMX-DHP) is an effective treatment for AE and prolongs the survival of AE of IPF patients [35,36,37]. PMX-DHP was originally introduced to manage sepsis or septic shock via neutrophil removal by the column. An early introduction of PMX-DHP within 3 days after disease onset was effective, especially for dermatomyositis [38]. Based on these reports, when we see severe inflammation with AE of IPF patients, we may consider PMX-DHP to be a possible therapeutic option. Recombinant human soluble thrombomodulin (rhTM), which has anti-inflammatory effects and mitigates the coagulation cascade, was also developed as a treatment for sepsis. In acute lung injury, there is both intense procoagulant activity and severe inflammation in the lung parenchyma [39]. Therefore, the mechanism of action of rhTMs suggests that it could be a plausible therapeutic agent for AEs of IPF. Several reports have shown that intravenous administration of rhTM for six consecutive days improved the survival rate of AEs of IPF patients [40,41,42,43,44]. Medical insurance does not cover these two therapies for AE of IPF, so we should discuss the use of such novel treatments with patients and their families very thoroughly. Tomioka et al. [45] reported that a case of AE of IPF improved with the use of nintedanib alone. When we see mild AE of IPF patients, antifibrotic agents may play a role during acute and chronic phases. In addition, high-flow nasal cannula (HFNC) is sometimes used for IPF with reduced tachypnea and improvements in minute ventilation [46]. In AEs of IPF patients, pharmacological treatment with HFNC can provide relief of dyspnea [47]. Future multicenter studies of HFNC are eagerly anticipated.

## 8. Prognosis

Acute exacerbation of IPF is usually associated with a poor prognosis. Natsuizaka et al. [48] reported that AE accounts for 40% of IPF deaths. The mean survival of AE of IPF is less than one year and 90-day mortality is approximately 50%. Therefore, the prevention of AE is crucial. Recent antifibrotic agents, especially nintedanib, were shown to prevent AE of IPF in an international clinical trial [49]. The subgroup analysis showed a 75% reduction of AE with nintedanib, especially among Japanese patients [50]. Pirfenidone combined with prednisolone and rhTM may improve survival in patients with AEs of IPF [51]. Therefore, the judicious use of these antifibrotic agents is likely to provide good prognoses and prevention of AEs of IPF patients [52].

In conclusion, IPF has a heterogeneous clinical course. Based on certain risk factors, physicians should carefully monitor patient status. An adequate diagnosis of AE using clinical information and chest HRCT permits early intervention for AEs of IPF. Acute exacerbation still has poor prognosis; therefore, novel management is required. 
